# Patients Aged 60 or Older with Surgically Treated Periprosthetic Acetabular Fractures Are at Similar Risk of Death at One Year Compared to Native Acetabular Fractures

**DOI:** 10.3390/jcm14186441

**Published:** 2025-09-12

**Authors:** Vera M. Stetzelberger, Julien Hirt, Iris F. Brouze, Simon D. Steppacher, Joseph M. Schwab, Moritz Tannast

**Affiliations:** 1 Department of Orthopaedic Surgery and Traumatology, Fribourg Cantonal Hospital, University of Fribourg, 1700 Fribourg, Switzerland; vera.stetzelberger@h-fr.ch (V.M.S.); julien.hirt@h-fr.ch (J.H.); iris.brouze@h-fr.ch (I.F.B.); josephmichael.schwab@h-fr.ch (J.M.S.); 2 Department of Orthopaedic Surgery and Traumatology, Inselspital Bern, University of Bern, 3010 Bern, Switzerland; simondamian.steppacher@insel.ch

**Keywords:** periprosthetic acetabular fracture, native acetabular fracture, geriatric trauma, total hip arthroplasty, mortality rates

## Abstract

**Background/Objectives:** Periprosthetic acetabular fractures in older adults are rare compared to other fractures of the hip. Nevertheless, their incidence is increasing due to an aging population with a growing number of total hip arthroplasties. Surgical management is complex, often involving frail patients, and one might expect an elevated risk of postoperative mortality. This study aimed to (1) determine the one-year mortality rate after operative treatment and (2) assess the perioperative complication rate of periprosthetic compared to native acetabular fractures. **Methods:** We identified 136 surgically treated acetabular fractures in patients ≥ 60 years (2012–2019): 31 periprosthetic and 105 native fractures. We performed case–control matching based on gender, frailty, osteoporosis, and comorbidities, yielding two matched cohorts (31 per group). (1) All patients were contacted, and the one-year mortality was assessed with Kaplan–Meier survivorship analysis before and after matching. (2) The perioperative complication rate was graded according to Sink. **Results:** (1) One-year mortality was 10% in the periprosthetic group vs. 20% in the native group (*p* = 0.19). After matching, no significant difference in the mortality rate was observed (10% vs. 23%, *p* = 0.17). (2) Overall complication rates were 61% in the periprosthetic group and 70% in the native group (*p* = 0.39). **Conclusions:** We observed a one-year mortality rate for periprosthetic acetabular fractures that was comparable to that of native acetabular fractures.

## 1. Introduction

The incidence of native acetabular fractures in older adult patients has risen over recent decades. In France, the reported incidence increased from 3.7 to 5.0 per 100,000 in the general population between 2006 and 2016, and reached 23.2 per 100,000 in patients older than 75 years [1]. Similar trends have been reported in other countries [2,3]. With rising life expectancy, projections estimate an overall incidence of 5.9 per 100,000 by 2030, and up to 32 per 100,000 in older patients [1]. Whereas in younger patients acetabular fractures usually result from high-energy trauma, such as falls from height or motor vehicle accidents, in older adults they most commonly occur after low-energy trauma, such as ground-level falls, and are frequently associated with osteoporotic bone. Typical fracture patterns in this population include anterior column, anterior wall, and associated anterior column with posterior hemitransverse fractures [4].

In parallel with increased life expectancy, the number of total hip arthroplasties (THAs) performed continues to grow, leading to a corresponding increase in periprosthetic acetabular fractures. Although still relatively rare with a reported incidence of 0.07% after primary THAs and 1.2% after revision procedures, orthopedic surgeons are likely to encounter these complex injuries more frequently in the coming years [5,6]. Due to their rarity, large series of patients with periprosthetic acetabular fractures are lacking, and little is known about their epidemiology or patient- and implant-related risk factors [7]. The surgical management of periprosthetic acetabular fractures is complex, often necessitating a combination of open reduction and internal fixation (ORIF) alongside revision arthroplasty. These procedures are technically demanding and are typically performed in medically frail, older adult patients, adding to their clinical complexity [8].

Given the higher complexity of the surgical management of periprosthetic acetabular fractures, one might expect a higher one-year mortality rate compared to native acetabular fractures. However, while the mortality of geriatric native acetabular fractures has been well reported [9,10,11,12], the mortality associated with periprosthetic acetabular fractures in older adult patients has not yet been evaluated.

Therefore, the aims of this study were as follows: (1) to determine the one-year mortality rate after operative treatment; and (2) to assess the perioperative complication rate of periprosthetic acetabular fractures compared to native acetabular fractures.

## 2. Materials and Methods

### 2.1. Study Design

This retrospective, comparative study (evidence level IV) was approved by our institutional review board (IRB). It compares one-year mortality and perioperative complication rates following surgical management of periprosthetic versus native acetabular fractures in a consecutive series of older adult patients treated at our Level 1 trauma center.

**Figure 1:**
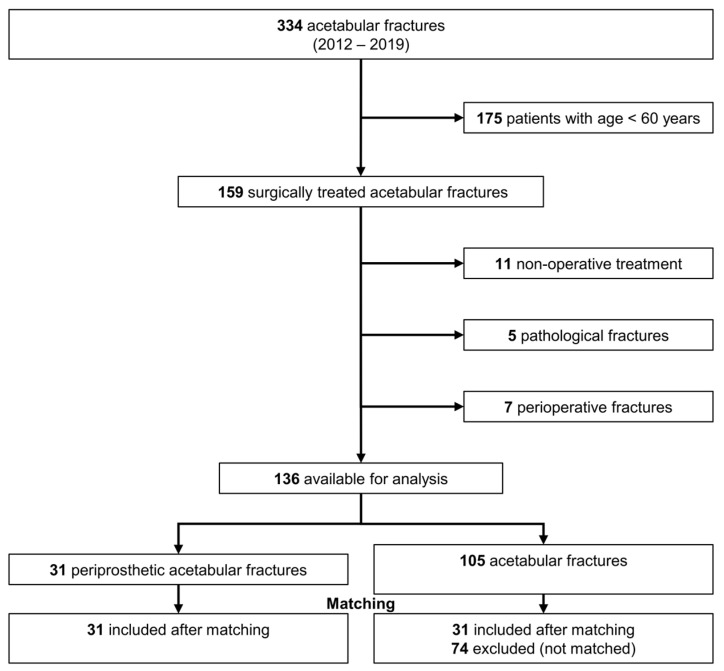
Flowchart demonstrating criteria for inclusion and reasons for exclusion.

We analyzed 334 consecutive patients who sustained an acetabular fracture at our Level-1 trauma center between January 2012 and December 2019. We excluded all patients younger than 60 years of age (n = 175), patients undergoing conservative treatment (n = 11), pathological fractures from metastatic cancer (n = 5), and perioperative fractures during total hip arthroplasty (n = 7). A total of 136 acetabular fractures were available for final analysis (Figure 1). Of those, 31 were periprosthetic (the periprosthetic acetabular fracture group) and 105 were native (the native acetabular fracture group).

The primary outcome was the one-year mortality. Day of death was determined by assessing the institutional electronic medical records, or by contacting family members, primary care providers, or nursing facilities. The secondary outcome was the perioperative complication rate, graded according to the classification of Sink et al. [13].

### 2.2. Surgical Indication and Management

The fracture was diagnosed by a LODOX Statscan (Lodox Systems, Johannesburg, South Africa) [14], conventional radiographs, and computed tomography (CT) scans. Periprosthetic and native acetabular fractures were classified according to Letournel [15]. Surgical indication was defined as a displacement exceeding 2 mm in any of the six radiographic acetabular lines described by Letournel and Judet [16]. The final decision regarding surgical intervention was made in collaboration with anesthesiology, based on the patient’s overall clinical condition and perioperative risk assessment. Surgical procedures were performed only after hemodynamic stabilization and optimization of cardiac and fluid status.

A single approach was preferred whenever possible. In cases of difficult reduction or where optimal access to the fracture required it, combined approaches were utilized. Anterior column and anterior wall fractures were usually treated using the pararectus approach [17], with/without a second approach. In posterior column and posterior wall fractures, we utilized a standard Kocher–Langenbeck approach, with or without osteotomy of the greater trochanter (“trochanter flip”). All surgeries were performed by senior specialist orthopedic trauma surgeons from our department.

### 2.3. Postoperative Management

Postoperatively, a standard intravenous antibiotic prophylaxis (1.5 g Zinacef^®^ GlaxoSmithKline AG, Vévey, Switzerland) was administered in all patients. Thromboprophylaxis was generally performed using low molecular weight heparin (Clexane^®^ Sanofi-Aventis SA, Geneva, Switzerland) until full weight bearing, unless modified by the internal medicine department. Based on the amount of intraoperative blood loss, tranexamic acid (Exacyl^®^ Sanofi-Aventis SA, Geneva, Switzerland) was used.

In both groups, regardless of whether ORIF was performed alone or in combination with THA, patients were generally allowed to partially weight bear (15 kg at least for 8 weeks). If partial weight bearing was not possible, wheelchair mobilization was prescribed until radiographic evidence of fracture healing. Postoperative mobilization was typically initiated on the first postoperative day under the supervision of a physical therapist, contingent upon the patient’s medical stability. When feasible, patients were transferred to specialized geriatric rehabilitation facilities to support early functional recovery. Routine clinical and radiographic follow-up was scheduled at 6 to 8 weeks, 3 months, 6 months, and annually thereafter. Patients who did not complete a full year of follow-up and were unable to attend in-person visits were classified as censored cases. No patients were lost to follow-up.

### 2.4. Assessment of Patient’s Characteristics

Clinical data were documented using electronic patient charts. American Society of Anesthesiologists (ASA) score [18,19], the Charlson Comorbidity Index (CCI) [20], and its age-adjusted variant (ACCI) [21], were used to evaluate patients’ baseline preoperative health status as these scores are recognized predictors of postoperative mortality. In addition, we systematically recorded the presence of cardiovascular disease [22,23], renal insufficiency [22], and malnutrition [23,24], as they are associated with higher mortality rates. We also considered the components of overlapping geriatric syndromes [25] by quantifying frailty using both the modified frailty index [26,27,28] and the 5-item modified frailty index [29,30]; sarcopenia using the psoas–lumbar–vertebra index [31]; osteoporosis using the cortical-thickness index [32]; and cachexia using the presence of malnutrition and body mass index. Overall, we considered a patient as “frail” if their modified frailty index was >0.27 [33].

Perioperative parameters such as intraoperative blood loss, number of packed red blood cell (pRBC) transfusions, and duration of surgery were gathered from electronic anesthesiology and operative records.

### 2.5. Statistical Analysis

Statistical analysis was performed using SPSS Version 25 (IBM Corporation, Armonk, NY, USA). We performed Kaplan–Meier survivorship analysis comparing both groups. The one-year mortality rate was further analyzed using the log-rank test. Hazard ratios (HRs) with corresponding 95% confidence intervals (CIs) were calculated. The mortality rates at different time points were compared using the Chi-squared test. To account for differences in important patient parameters (demography, comorbidities, and overlapping components of geriatric syndromes) and allow for a better comparability, we performed case–control matching. The random matching of cases was completed based on gender, modified frailty index > 0.27 indicating frailty [26,27,28,34,35,36], cortical thickness index < 0.40 [32] indicating osteoporosis, and ACCI > 5 [21]. After the matching process two comparable groups of 31 patients were available for further analysis.

Normality was tested using the Kolmogorov–Smirnov test. Normally distributed variables were compared using the Student’s *t*-test, and non-normally distributed variables using the Mann–Whitney test. Categorical variables analyzed using the Chi-squared test for large sample sizes, and Fisher’s exact test was applied in small sample sizes. Results were reported as mean ± standard deviation with range in parentheses for continuous values, and as absolute numbers with percentage in parentheses for categorical values.

## 3. Results

The mean age of patients with periprosthetic and native acetabular fractures was 80 ± 9 years (64–97) and 77 ± 9 years (61–94), respectively (*p* = 0.07; Table 1). In the periprosthetic acetabular fracture group, 26% of patients were male, compared to 69% in the native acetabular fracture group (*p* < 0.001). No significant difference in preoperative health status and comorbidities were reported between the two groups except sarcopenia (*p* = 0.02) and osteoporosis (*p* < 0.001), which were both more prevalent in the periprosthetic acetabular fracture group. A ground-level fall was the mechanism of injury in 90% of patients with periprosthetic fractures compared to 77% in patients with native fractures (*p* = 0.05; Table 2).

In both groups, 35% of patients underwent surgery within 48 h of hospital admission (Table 3).

ORIF was performed in 11 (35%) periprosthetic and 88 (84%) native fractures. A total of 20 patients (65%) with periprosthetic fractures underwent THA revision with concomitant ORIF compared to 17 patients (16%) with native fractures (Table 4). Bone grafting was used in 16 (52%) periprosthetic fractures and 42 (40%) native fractures (*p* = 0.10).

### 3.1. One-Year Mortality

**Figure 2:**
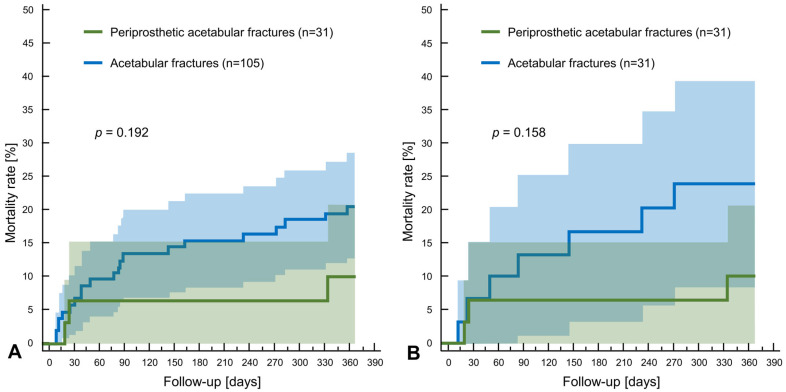
The Kaplan–Meier curve shows the overall one-year mortality rate of periprosthetic and control acetabular fractures before (**A**) and after (**B**) matching.

The one-year mortality was 10% (n = 3) for periprosthetic fractures (HR 0.53; CI 95% 0.21–1.37; *p* = 0.19), compared to 20% (n = 21) for native acetabular fractures (HR 1.87; CI 95% 0.73–4.79) (Figure 2A; Table 5). After matching, the one-year mortality was 10% in the periprosthetic group (HR 0.41; CI 95% 0.12–1.42; *p* = 0.17), compared to 23% (HR 2.45; CI 95% 0.71–8.50) in the native group. In the periprosthetic group, no patient died during their hospital stay, whereas 3.8% (n = 4) from the native group died (*p* = 0.19).

### 3.2. Complications

The overall complication rate was 61% in the periprosthetic acetabular fracture group, compared to 70% in the native acetabular fracture group (*p* = 0.39; Table 6). Minor complications (Grade I–II) were observed in 58% of patients in the periprosthetic group versus 68% in the native group (*p* = 0.33). Major complications (Grade III–IV) accounted for 13% in both groups.

No significative differences were observed in the rates of Grade I, II, III, and IV complications according to the grading system of Sink et al. [13]. The most frequent complication was postoperative anemia requiring an RBC transfusion.

Among Grade III complications, three patients developed postoperative hematomas, two of which required surgical revision for evacuation. One patient in the periprosthetic fracture group had a deep infection treated with debridement and antibiotics. Three patients in the native fracture group developed deep infections: two were treated with revision surgery including debridement and antibiotics, and one required a two-staged revision surgery with an exchange of the THA. One patient developed a thrombosis of the triceps surae veins, which was managed with anticoagulation therapy. One patient had a secondary dislocation of the acetabular dome, which was treated with reoperation via pararectus approach and repositioning of the fragment using a Locking Compression Plate (LCP) plate and two 3.5 mm screws.

Regarding Grade IV complications, two patients had nerve injuries in the periprosthetic group. One developed femoral nerve compression with M3 paresis (Medical Research Council Scale [37]) of the quadriceps muscle due to a hematoma, which required a fasciotomy and hematoma evacuation, resulting in full recovery at one year. The second sustained peroneal nerve paresis, with complete recovery at one year. One patient had a segmental pulmonary embolism, which was treated with oral anticoagulation therapy. In the native acetabular group, two patients sustained vessel injuries. One had a lesion of the superior glutal artery resulting in compartment syndrome, which required a revision surgery with vessel ligation and hematoma evacuation. The other had an injury to a branch of the internal iliac artery, which was managed with a tamponade and drainage. Five patients experienced nerve injuries. One had an obturator nerve lesion with complete recovery at one year. Four had sciatic nerve paresis: one achieved complete recovery, one had complete motor recovery but persistent paresthesia, and two had partial recovery, still requiring a Heidelberg splint at one year.

Four patients, all in the native acetabular fracture group, died during their hospital stay. Two deaths were due to implant-associated infections. The other two resulted from decompensation of pre-existing comorbidities: one from hepatic encephalopathy secondary to cirrhosis, and the other from respiratory decompensation secondary to interstitial lung disease.

## 4. Discussion

We found that the one-year mortality rate following surgically treated periprosthetic acetabular fractures in older adult patients did not significantly differ from that of native acetabular fractures. The one-year mortality rate for native acetabular fractures was 20%, consistent with rates reported in the literature, which range from 5% to 26% depending on patient age, comorbidities, and treatment modality [9,10,12,38]. In contrast, the one-year mortality rate for periprosthetic acetabular fractures was 10%. Notably, in our study, no deaths occurred in the periprosthetic acetabular fracture group during the initial hospitalization, whereas 19% of deaths in the native acetabular fracture group were in-hospital. In both groups, most deaths occurred in patients older than 80 years, underscoring the impact of advanced age on postoperative survival.

Data on mortality in patients with periprosthetic acetabular fractures remains sparse, and to our knowledge, this is the first study to report a one-year mortality rate specifically for this group. Selmene et al. reported one death (5%) within 15 days after surgery due to stroke, while Peterson et al. described one death (9%) related to intrapelvic vascular injury [5,39]. In the small series of Rommens et al. (n = 6), no in-hospital deaths occurred, although one patient died after 82 weeks [40].

Interestingly, despite the increased surgical complexity typically associated with periprosthetic acetabular fractures, these injuries were not associated with higher one-year mortality compared to native acetabular fractures. This finding is in line with prior literature suggesting that apparently complex fractures are not automatically associated with a higher mortality risk. A previous matched cohort study comparing older adult patients with acetabular fractures versus proximal femur fractures reported a significantly lower one-year mortality in the acetabular fracture group (18% vs. 36%). The length of hospital stay and postoperative weight-bearing restrictions also did not differ between the two groups [41].

Patients with surgically treated periprosthetic acetabular fractures in our series did not experience a higher rate of perioperative complications compared to those with native acetabular fractures. In both groups, most complications were classified as minor (Sink Grade I–II), with postoperative anemia accounting for the vast majority. This is consistent with previously reported complication profiles in older adult patients undergoing surgery for acetabular fractures [41]. Complication rates after acetabular fracture surgery in patients over 65 years have been reported at approximately 37%, with infection accounting for 11% and venous thromboembolism for 9% [42]. For periprosthetic acetabular fractures, Selmene et al. reported dislocation in 30% of cases and infection in 20% [39]. Frietman et al. reported a 15% rate of severe postoperative complications in surgically treated native acetabular fractures, including permanent iatrogenic sciatic nerve palsy in 8%, deep wound infection in 5%, and two cases of intraarticular screws [43]. Ljungdhal et al. reported an overall complications rate of 38% in patients older than 70 years [12].

As expected, the predominant mechanism of injury in both groups was low-energy trauma, most commonly a fall from standing height. This reflects the inclusion of patients aged 60 years and older, in whom acetabular fractures are typically fragility-related. In contrast, younger patients more often sustain these injuries through high-energy mechanisms such as motor vehicle accidents or sports accidents [44].

Although functional outcomes were not the focus of the present study, they remain an important consideration, particularly for older adult trauma patients, whose primary goal is often to regain independency. Functional outcome data in the literature are limited. Selmene et al. reported a mean Harris Hip Score of 75.5 at final follow-up, with 70% of patients regaining their pre-fracture level of autonomy [39]. Frietman et al. found a mean modified Harris Hip Score of 87 in patients with native acetabular fractures treated with ORIF alone, compared to 74 in those treated with ORIF and total hip arthroplasty [43].

This study has several limitations. First, the original groups were unequal in size, and there was an imbalance in sex distribution and the prevalence of sarcopenia and osteoporosis. However, a 1:1 matched case–control analysis was performed, and after matching, no significant differences remained between the groups in the baseline characteristics.

Second, although we controlled for key geriatric risk factors such as frailty, osteoporosis, and comorbidity burden, unmeasured variables, such as cognitive status, socioeconomic support, or functional capacity, may have influenced outcomes.

Third, our study excluded patients who underwent non-operative treatment. This limits the generalizability of our findings to all older adult patients with acetabular fractures.

Fourth, we did not assess short- and long-term recovery following surgical treatment of native versus periprosthetic acetabular fractures. The use of patient-reported outcome measures, such as the EQ-5D or hip-specific scores (International Hip Outcome Tool-12 (iHOT-12), Harris Hip Score, or Western Ontario and McMaster Universities Osteoarthritis Index (WOMAC)), could help detect differences in postoperative functional recovery and satisfaction between the two groups.

Five, this was a single-center, retrospective study conducted at a specialized Level 1 trauma center. While this setting allowed for access to comprehensive clinical documentation, the creation of a national registry of native and periprosthetic acetabular fractures would provide greater generalizability of the results.

## 5. Conclusions

Although the operative treatment of periprosthetic acetabular fractures is complex and requires extensive surgery, we observed a one-year mortality rate comparable to native acetabular fractures in our study. Similarly, the overall perioperative complication rates were comparable between the two groups. These findings support the safety of operative management for periprosthetic acetabular fractures in experienced centers.

## Figures and Tables

**Table 1:** Basic demographics, preoperative comorbidities, and elements of overlapping geriatric syndromes.

	Overall Cohort (n = 136)			Matched Cohort (n = 62)		
Parameter	Periprosthetic Acetabular Fracture Group (n = 31)	Native Acetabular Fracture group (n = 105)	*p*-Value	Periprosthetic Acetabular Fracture Group (n = 31)	Native Acetabular Fracture Group (n = 31)	*p*-Value
Demographic						
Age (years)	80 ± 9 (64–97)	77 ± 9 (61–94)	0.07	80 ± 9 (64–97)	80 ± 9 (62–94)	0.97
Sex (% men of all hips)	8 (26)	72 (69)	**<0.001**	8 (26)	8 (26)	1
Side (% right of all hips)	15 (48)	58 (55)	0.50	15 (48)	16 (52)	0.80
Body mass index (kg/m^2^)	25 ± 7 (17–49)	25 ± 4 (17–48)	0.99	25 ± 7 (17–49)	24 ± 4 (17–32)	0.53
Nursing home resident	5 (16)	15 (14)	0.83	5 (16)	7 (23)	0.52
Preoperative health status and comorbidities						
ASA score	2.9 ± 0.78 (2–4)	2.8 ± 0.65 (1–4)	0.92	2.9 ± 0.78 (2–4)	3.0 ± 0.5 (2–4)	0.62
ACCI score	5.6 ± 2.2 (2–10)	5.8 ± 3.1 (2–15)	0.80	5.6 ± 2.2 (2–10)	6.8 ± 3 (3–14)	0.24
Patients with cardiovascular disease	11 (35)	37 (35)	0.98	11 (35)	10 (32)	0.79
Patients with renal failure	6 (19)	19 (19)	0.87	6 (19)		
Patients with liver cirrhosis	1 (3)	9 (9)	0.31	1 (3)	2 (6)	0.56
Components of overlapping geriatric syndromes						
Frail patient ^a^	9 (29)	23 (22)	0.67	9 (29)	7 (23)	0.56
Modified frailty index	0.16 ± 0.12 (0–0.5)	0.15 ± 0.12 (0–0.6)	0.73	0.16 ± 0.12 (0–0.5)	0.16 ± 0.11 (0–0.45)	0.91
5-item modified frailty index	1.3 ± 0.9 (0–3)	1.4 ± 0.9 (0–4)	0.83	1.3 ± 0.9 (0–3)	1.6 ± 1 (0–4)	0.37
Sarcopenia ^b^	9 (29)	13 (12)	**0.02**	9 (29)	6 (19)	0.34
Patients with malnutrition	4 (13)	10 (10)	0.37	4 (13)	6 (19)	0.49
BMI < 20 kg/m^2^ (Cachexia)	6 (19)	10 (10)	0.14	6 (19)	6 (19)	1
Osteoporosis ^c^	10 (32)	5 (5)	**<0.001**	10 (32)	4 (13)	0.07

^a^ Frail is defined as a modified frailty index > 0.27; ^b^ Sarcopenia is defined as a psoas–lumbar–vertebra index PLVI < 0.84; ^c^ Osteoporosis is defined as cortical thickness index < 0.40. ACCI: Age-adjusted Charlson Comorbidity Index; ASA score: American Society of Anesthesiologists score; BMI: Body Mass Index. Bold values indicate *p* < 0.05.

**Table 2:** Injury characteristics of both groups.

	Overall Cohort (n = 136)			Matched Cohort (n = 62)		
Parameter	Periprosthetic Acetabular Fracture Group (n = 31)	Native Acetabular Fracture Group (n = 105)	*p*-Value	Periprosthetic Acetabular Fracture Group (n = 31)	Native Acetabular Fracture Group (n = 105)	*p*-Value
Mechanism of trauma						
Ground-level fall	28 (90)	81 (77)	0.05	28 (90)	28 (90)	1
Fall from height > 2 m	0 (0)	4 (4)	0.27	0 (0)	1 (3.2)	0.32
Sports accident	2 (6.5)	13 (12)	0.14	2 (6.5)	2 (6.5)	1
Motor vehicle accident	1 (3.2)	7 (7)	0.48	1 (3.2)	0 (0)	0.32
Associated injuries						
None	27 (87)	69 (66)	0.19	27 (87)	25 (81)	0.49
Pelvic fracture	0 (0)	6 (6)	0.17	0 (0)	2 (2)	0.145
Spine injury	0 (0)	1 (1)	0.59	0 (0)	0 (0)	1
Traumatic hip/THA dislocation	1 (3)	12 (9)	0.06	1 (3)	1 (3)	1
Upper extremity fracture	3 (10)	6 (6)	0.42	3 (10)	2 (6.5)	0.62
Lower extremity fracture	0 (0)	3 (3)	0.35	0 (0)	2 (2)	0.228
Subdural or intracranial bleeding	0 (0)	5 (5)	0.22	0 (0)	2 (6.5)	0.393
Cerebral concussion	0 (0)	2 (2)	0.44	0 (0)	2 (6.5)	0.981
Visceral organ damage	0 (0)	1 (1)	0.59	0 (0)	0 (0)	0.312

THA: Total hip arthroplasty.

**Table 3:** Perioperative parameters and postoperative weight-bearing status before and after matching.

	Overall Cohort (n = 136)			Matched Cohort (n = 62)		
Parameter	Periprosthetic Acetabular Fracture Group (n = 31)	Native Acetabular Fracture Group (n = 105)	*p*-Value	Periprosthetic Acetabular Fracture Group (n = 31)	Native Acetabular Fracture Group (n = 31)	*p*-Value
Time to surgery (days)	7 ± 9 (0–37)	6 ± 6 (0–33)	0.51	7 ± 9 (0–37)	7 ± 9 (0–33)	0.55
Surgery duration (min)	190 ± 80 (48–375)	181 ± 66 (65–450)	0.54	190 ± 80 (48–375)	173 ± 63 (100–300)	0.42
Intraoperative blood loss (mL)	724 ± 348 (200–2000)	1052 ± 812 (200–4500)	0.06	724 ± 348 (200–2000)	968 ± 732 (200–3400)	0.30
RBC transfusion	16 (52)	65 (62)	0.28	16 (52)	21 (68)	0.20
Tranexamic acid (% yes)	13 (42)	62 (59)	0.05	13 (42)	16 (52)	0.31
Tranexamic acid dose (mg)	403 ± 490 (0–1000)	650 ± 562 (0–2000)	0.03	403 ± 490 (0–1000)	552 ± 557 (0–2000)	0.31
Length of hospital stay (days)	12 ± 6 (4–29)	11 ± 6 (3–42)	0.69	12 ± 6 (4–29)	11 ± 6 (4–27)	0.64
Wheelchair mobilization	17 (55)	49 (47)	0.43	17 (55)	13 (42)	0.31
Partial weight bearing	13 (42)	52 (50)	0.46	13 (42)	18 (58)	0.21
Days of partial weight bearing (days)	57 ± 8 (42–84)	61 ± 14 (7–90)	0.18	57 ± 8 (42–84)	61 ± 17 (14–84)	0.66
Full weight bearing	1 (3)	4 (4)	0.88	1 (3)	0	0.32

RBC transfusion: Red blood cell transfusion.

**Table 4:** Fracture pattern and surgical approaches for both study groups.

Parameter	Periprosthetic Acetabular Fractures (n = 31)		Native Acetabular Fractures (n = 105)	
Fracture pattern	Simple fracture type	14 (45)	Simple fracture type	17 (16)
	Anterior wall	0 (0)	Anterior wall	4 (4)
	Anterior column	4 (13)	Anterior column	3 (3)
	Posterior wall	0 (0)	Posterior wall	7 (7)
	Posterior column	1 (3)	Posterior column	3 (3)
	Transverse	9 (29)	Transverse	0 (0)
	Associated fracture type	17 (55)	Associated fracture type	88 (84)
	Posterior column and posterior wall	0 (0)	Posterior column and posterior wall	0 (0)
	T-shaped	11 (35)	T-shaped	4 (4)
	Anterior column and posterior hemitransverse	5 (16)	Anterior column and posterior hemitransverse	49 (47)
	Transversed posterior wall	0 (0)	Transversed posterior wall	3 (3)
	Both columns	1 (3)	Both columns	32 (30)
Surgical approach	Single approach	25 (81)	Single approach	87 (83)
	Pararectus	11 (35)	Pararectus	66 (63)
	Modified Kocher–Langenbeck	3 (10)	Modified Kocher–Langenbeck	1 (1)
	Transgluteal	8 (26)	Transgluteal	2 (2)
	Trochanteric flip	3 (10)	Trochanteric flip	12 (11)
	Stoppa	0	Stoppa	6 (6)
	Two combined approaches	6 (19)	Two combined approaches	18 (17)
	Pararectus and trochanteric flip	0 (0)	Pararectus and trochanteric flip	7 (7)
	Pararectus and transgluteal	3 (10)	Pararectus and transgluteal	1 (1)
	Pararectus and anterior	0 (0)	Pararectus and anterior	2 (2)
	Pararectus and Smith–Peterson	1 (3)	Pararectus and Smith–Peterson	3 (2)
	Transgluteal and Kocher–Langenbeck	1 (3)	Transgluteal and Kocher–Langenbeck	4 (3)
	Smith–Peterson and Stoppa	1 (3)	Smith–Peterson and Stoppa	1 (1)
Type of surgery	ORIF	11 (35)	ORIF	88 (84)
	ORIF and revision THA	20 (65)	ORIF and THA	17 (16)
	Bone grafting	16 (52)	Bone grafting	42 (40)
	Allograft	14 (45)	Allograft	30 (29)
	Autograft	2 (6)	Autograft	12 (11)

ORIF = Open reduction and internal fixation; THA = Total hip arthroplasty.

**Table 5:** Mortality at different time points and one-year mortality for different age subgroups.

	Overall Cohort (n = 136)			Matched Cohort (n = 62)		
Parameter	Periprosthetic Acetabular Fracture Group (n = 31)	Native Acetabular Fracture Group (n = 105)	*p*-Value	Periprosthetic Acetabular Fracture Group (n = 31)	Native Acetabular Fracture Group (n = 31)	*p*-Value
Mortality time points						
In-hospital	0 (0)	4 (4) (0–8)	0.19	0 (0)	1 (3) (0–9)	0.32
Thirty days	2 (6) (0–15)	6 (6) (1–10)	0.88	2 (6) (0–15)	2 (6) (0–15)	1
Two months	2 (6) (0–15)	10 (13) (4–15)	0.60	2 (6) (0–15)	3 (10) (0–21)	0.64
Four months	2 (6) (0–15)	14 (15) (7–20)	0.30	2 (6) (0–15)	4 (13) (1–25)	0.39
Six months	2 (6) (0–15)	16 (15) (8–22)	0.21	2 (6) (0–15)	5 (16) (3–30)	0.23
Eight months	2 (6) (0–15)	17 (16) (9–23)	0.17	2 (6) (0–15)	6 (19) (6–35)	0.13
Ten months	2 (6) (0–15)	19 (18) (10–26)	0.12	2 (6) (0–15)	7 (23) (8–39)	0.07
One year	3 (10) (0–21)	21 (20) (13–28)	0.19	3 (10) (0–21)	7 (23) (8–39)	0.17
One year mortality in different age categories	*p* = 0.75	*p* = 0.004		*p* = 0.75	*p* = 0.60	
≤80 years	1 (8) (0–24)	7 (12) (4–19)	0.73	1 (8) (0–24)	4 (27) (4–50)	0.20
>80 years	2 (11) (0–26)	14 (35) (20–50)	0.07	2 (11) (0–26)	3 (10) (0–41)	0.65

**Table 6:** Complications for the two study groups according to Sink et al. [13].

Parameter	Periprosthetic Acetabular Fracture Group (n = 31)	Native Acetabular Fracture Group (n = 105)	*p*-Value
No complications	12 (39)	32 (30)	0.39
Minor complications (grade I and II)	18 (58)	71 (68)	0.33
Major complications (grade III and higher)	4 (13)	14 (13)	0.95
Grade I	0	3 (3)	0.34
Postoperative anemia without transfusion	0	3 (3)	0.50
Grade II	18 (58)	68 (65)	0.70
Postoperative anemia with transfusion	17 (55)	67 (64)	0.34
Postoperative delirium	2 (6)	17 (16)	0.17
Pneumonia	0	1 (1)	0.59
Superficial wound infection	0	1 (1)	1
Paralytic ileus	0	1 (1)	0.59
Grade III	2 (6)	6 (6)	0.88
Hematoma	1 (3)	2 (2)	0.66
Deep infection	1 (3)	3 (3)	0.34
Deep vein thrombosis	0	1 (1)	0.59
Secondary dislocation	0	1 (1)	0.59
Grade IV	3 (10)	8 (8)	0.71
Nerve injury	2 (6)	5 (5)	0.70
Vessel injury	0	2 (2)	0.44
Urosepsis	0	1 (1)	0.59
Pulmonary embolism	1 (3)	1 (1)	0.35
Grade V	0	4 (4)	0.27
In-hospital death	0	4 (4)	0.27

One patient could be affected from several complications of the same grade according to Sink et al. [13].

## Data Availability

Data is contained within the article.

## Abbreviations

The following abbreviations are used in this manuscript:

ACCI	Age-adjusted Charlson Comorbidity Index
BMI	Body Mass Index
CCI	Charlson Comorbidity Index
CI	Confidence Interval
CT	Computed Tomography
HR	Hazard ratio
iHOT-12	International Hip Outcome Tool-12
IRB	Institutional Review Board
LCP	Locking Compression Plate
ORIF	Open Reduction and Internal Fixation
PLVI	Psoas Lumbar Vertebra Index
RBC	Red Blood Cell
THA	Total Hip Arthroplasty
WOMAC	Western Ontario and McMaster Universities Osteoarthritis Index
